# Manganese therapy for dyslipidemia and plaque reversal in murine models

**DOI:** 10.1093/lifemeta/load040

**Published:** 2023-10-26

**Authors:** Yawei Wang, Xin Feng, Wenjing Zhou, Runze Huang, Yating Hu, Hui Hui, Jie Tian, Xiao Wang, Xiao-Wei Chen

**Affiliations:** State Key Laboratory of Membrane Biology, Peking University, Beijing 100871, China; PKU-THU Joint Center for Life Sciences, Peking University, Beijing 100871, China; CAS Key Laboratory of Molecular Imaging, Institute of Automation, Chinese Academy of Sciences, Beijing 100190, China; Beijing Key Laboratory of Molecular Imaging, Beijing 100190, China; School of Artificial Intelligence, University of Chinese Academy of Sciences, Beijing 100080, China; State Key Laboratory of Membrane Biology, Peking University, Beijing 100871, China; Institute of Molecular Medicine, College of Future Technology, Peking University, Beijing 100871, China; State Key Laboratory of Membrane Biology, Peking University, Beijing 100871, China; Institute of Molecular Medicine, College of Future Technology, Peking University, Beijing 100871, China; State Key Laboratory of Membrane Biology, Peking University, Beijing 100871, China; Institute of Molecular Medicine, College of Future Technology, Peking University, Beijing 100871, China; CAS Key Laboratory of Molecular Imaging, Institute of Automation, Chinese Academy of Sciences, Beijing 100190, China; Beijing Key Laboratory of Molecular Imaging, Beijing 100190, China; School of Artificial Intelligence, University of Chinese Academy of Sciences, Beijing 100080, China; CAS Key Laboratory of Molecular Imaging, Institute of Automation, Chinese Academy of Sciences, Beijing 100190, China; Beijing Key Laboratory of Molecular Imaging, Beijing 100190, China; State Key Laboratory of Membrane Biology, Peking University, Beijing 100871, China; Institute of Molecular Medicine, College of Future Technology, Peking University, Beijing 100871, China; State Key Laboratory of Membrane Biology, Peking University, Beijing 100871, China; Institute of Molecular Medicine, College of Future Technology, Peking University, Beijing 100871, China; PKU-THU Joint Center for Life Sciences, Peking University, Beijing 100871, China

## Abstract

**Figure:**
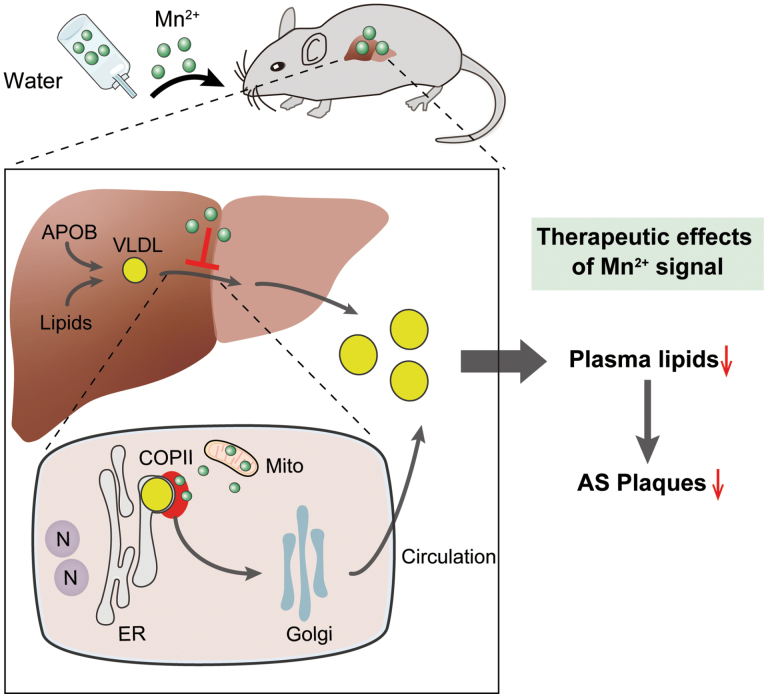
Precise control of circulating lipid levels is vital in both health and disease. We recently uncovered that bulk lipids, transported by lipoproteins, enter the circulation initially via the coat protein complex II (COPII) in a condensation-dependent manner. Divalent manganese, acting as a signaling messenger, selectively controls COPII condensation to regulate lipid homeostasis *in vivo*. Here, we present evidence for a manganese-based therapy in murine models of hypolipidemia and hyperlipidemia, aided by advanced *in vivo* multimodal imaging of atherosclerosis. Dietary titration of manganese supply enables tailored control of circulating lipid levels in whole animals, with no apparent toxicity. Strikingly, elevating the manganese signal through diets could not only effectively treat pathological hyperlipidemia but also further achieve significant reversal of atherosclerotic plaques. Hence, the study provides critical proof-of-principle for a novel therapy for deadly cardiovascular diseases with a potentially broad impact.

Precise control of circulating lipid levels is vital in both health and disease. We recently uncovered that bulk lipids, transported by lipoproteins, enter the circulation initially via the coat protein complex II (COPII) in a condensation-dependent manner. Divalent manganese, acting as a signaling messenger, selectively controls COPII condensation to regulate lipid homeostasis *in vivo*. Here, we present evidence for a manganese-based therapy in murine models of hypolipidemia and hyperlipidemia, aided by advanced *in vivo* multimodal imaging of atherosclerosis. Dietary titration of manganese supply enables tailored control of circulating lipid levels in whole animals, with no apparent toxicity. Strikingly, elevating the manganese signal through diets could not only effectively treat pathological hyperlipidemia but also further achieve significant reversal of atherosclerotic plaques. Hence, the study provides critical proof-of-principle for a novel therapy for deadly cardiovascular diseases with a potentially broad impact.

## Dear Editor,

Cardiovascular diseases (CVDs) and related metabolic disorders continue to rank as the leading cause of human mortality, causing >20 million deaths per year worldwide. Hyperlipidemia, or elevated circulating lipid levels, stands out as the primary risk factor of CVDs, notably through the inception of atherosclerosis [1]. The formation of atherosclerotic plaques is a protracted process. However, unstable plaques are prone to rupture and hemorrhage, initiating acute thrombosis that may trigger life-threatening myocardial infarction or stroke [2]. Of note, atherosclerotic plaques in their nascent stages frequently remain clinically “silent”, and, therefore, often evade patient awareness. Given this context, effective means to safely reverse the existing atherosclerotic plaques assume great therapeutic significance [3]. Regrettably, such approaches remain to be established to date, representing a major unmet medical need.

Due to their hydrophobic nature, bulk lipids including triglycerides and cholesterol are ferried into the circulation in the form of specialized lipoproteins. Apolipoprotein B (APOB) serves as the major structural protein for the outbound lipoproteins, namely chylomicrons (CM) secreted from the small intestine and very low-density lipoprotein (VLDL) released from the liver. In contrast to conventional secretory proteins, lipoproteins exhibit distinct attributes of high abundance, large size, and complex biochemical composition, all governed by metabolic signals [4]. While these lipid carriers embark on the secretory pathway from the endoplasmic reticulum (ER) via the universal coat protein complex II (COPII) machinery, one may speculate that unique regulatory mechanisms may evolve to precisely control lipid supply via lipoprotein secretion. Consistent with the idea, mutations in the human *SAR1B*, encoding one of the COPII-operating GTPases, cause the rare disease chylomicron retention disease [5]. The affected patients exhibit a particular defect in diminished secretion of CM from the intestinal epithelial cells. Consequently, this defect leads to malabsorption of dietary fats, growth retardation, and failure to thrive, representing the other end of the spectrum of systemic lipid disorder, hypolipidemia [5].

We have recently reported a specialized lipoprotein export program that is characterized by high selectivity and quantitative plasticity, potentially leading to novel means of modulating systemic lipid homeostasis [6, 7]^,^. SAR1B GTPase, paired with the cargo receptor surfeit locus protein 4 (SURF4), initiates the ER export of lipoproteins via COPII-coated vesicles [8]. Moreover, SURF4 also partners with biogenic enzymes such as the ER phospholipid scramblase transmembrane protein 41B (TMEM41B) to couple the production and transport of lipoproteins [9], further highlighting the central and integrative role of the receptor-­mediated lipoprotein ER export program. We further discovered that the COPII machinery employs self-constrained condensation to balance dynamic and coat assembly, thereby maximizing the efficiency of lipoprotein export. Of note, divalent manganese, which can be mobilized from mitochondria stores, serves as a signal messenger to quantitatively tune COPII condensation-based functions, thus enabling a unique bell-shaped regulation on lipoprotein secretion and preventing dyslipidemia [10]. Moreover, dietary manganese can be effectively accumulated in the mitochondria-packed hepatocytes. Therefore, one may hypothesize that modulating manganese signal to target condensation-dependent lipid delivery by hepatic COPII may provide a therapeutic regimen for tailored lipid management, thereby effectively treating dyslipidemia and related cardiometabolic diseases.

To test the therapeutic potential of Mn^2+^ in murine models, we first sought to estimate the safe doses of orally supplied Mn^2+^ by determining the LD_50_ (the median lethal dose) of Mn^2+^ administration in wild-type C57BL/6J mice. A single oral gavage was administered at doses up to 1 g/kg body weight (Supplementary Fig. S1a), and survival was monitored for 1 week. The LD_50_ was calculated to be ~286 mg/kg body weight. Importantly, neither paralysis nor lethality was observed in mice receiving Mn^2+^ doses of 250 mg/kg body weight or lower. We also assessed the kinetics of orally supplied Mn^2+^ in mice. Baseline blood samples were collected at time zero, followed by a single oral administration of Mn^2+^ (30 mg/kg body weight). Blood samples were collected at 0.25-, 1-, 2-, 4-, 8- and 24-h post-administration to determine the Mn^2+^ concentration using inductively coupled plasma-mass spectrometry. The calculated half-life of orally supplied Mn^2+^ in the blood was 2.18 h (Supplementary Fig. S1b). We also examined the hepatic content of Mn^2+^ during this process and observed a rapid elevation within 4 h (Supplementary Fig. S1c), reflecting a quick distribution of exogenous Mn^2+^ into the liver as previously reported [11]. Hence, the hepatic enrichment of exogenous Mn^2+^ supplied with diet or drink enables our further investigation on lipid delivery from the liver.

The above results led us to first design Mn^2+^ administration to wild-type mice on normal diets by daily oral gavage of Mn^2+^ at different doses for 30 days (Supplementary Fig. S1d). Even at the highest dose given (40 mg/kg body weight), Mn^2+^ administration did not appear to alter body weight (Supplementary Fig. S1e). Remarkably, after one month, the blood lipid levels in wild-type mice exhibited a bell-shaped response to the Mn^2+^ dose administered (Supplementary Fig. S1f and g). Profiling of plasma lipids using size exclusion chromatography further revealed a bell-shaped regulation by Mn^2+^ on atherogenic lipoproteins including VLDLs and low-density lipoprotein (LDLs) (Supplementary Fig. S1h–k). While mice receiving Mn^2+^ at 5 mg/kg body weight displayed the highest lipid levels, levels of LDL-cholesterol (LDL-C, the major atherogenic lipid species) were decreased by ~50% in mice receiving the highest dose of 40 mg/kg body weight. Accordingly, similar bell-shaped responses to administered Mn^2+^ were observed in plasma APOB levels (Supplementary Fig. S1l and m). However, circulating levels of the conventional secretory proteins such as albumin remained unaltered, confirming the selective impact of the Mn^2+^ messenger on lipoprotein secretion over general secretion (Supplementary Fig. S1m). Taken together, these results provided evidence for the *in vivo* efficacy of Mn^2+^ treatment in blood lipid regulation.

To systematically evaluate the specificity and safety of Mn^2+^ administration in blood lipid control *in vivo*, we conducted transcriptomic analysis (RNA-Seq) to analyze mRNA profiles of liver samples from mice with Mn^2+^ administration (0, 5, and 20 mg/kg body weight). Principal component analysis (PCA) of gene expression profiles showed little separation of all the samples receiving different Mn^2+^ doses, indicating minimal changes in gene expression in the livers upon elevation of the Mn^2+^ signal (Supplementary Fig. S2a). Gene set enrichment analysis showed moderately increased expression of genes in respiration chain complex assembly and decreased expression of genes in metal ion transport in the liver from mice receiving Mn^2+^ of 20 mg/kg body weight compared to control mice receiving mock administration, probably owing to metabolic adaptations to exogenous manganese supply (Supplementary Fig. S2b–e). Meanwhile, little alteration was observed in stress-related pathways including ER stress or mitochondria function. Accordingly, immunoblotting analysis showed no obvious changes in ER stress or Golgi integrity, although the levels of the manganese sensor transmembrane protein 165 (TMEM165) were reduced by Mn^2+^ administration as expected (Supplementary Fig. S2f). Taken together, the transcriptome and biochemical analysis suggest a grossly normal hepatic function in mice with Mn^2+^ administration with the dosage that produced lipid-lowering effects in this study.

Histology analysis also revealed little alteration in liver morphology, though mild hepatic lipid accumulation was observed in mice receiving Mn^2+^ administration at the highest dose (Supplementary Fig. S2g−i). Furthermore, no signs of immune cell infiltration or fibrosis were detected in all samples. Along the same line, plasma alanine aminotransferase (ALT) and aspartate aminotransferase (AST) remained the same in all samples, indicating the absence of liver damage even in mice receiving Mn^2+^ administration at the highest dose (40 mg/kg body weight) (Supplementary Fig. S2j). Further, most tissues in mice receiving Mn^2+^ exhibited normal histology indistinguishable from those in control mice (Supplementary Fig. S3a). Circulating ­levels of creatine kinase remained similar to those in control mice (Supplementary Fig. S3b), confirming overall healthy states in Mn^2+^-administered mice.

**Figure 1:**
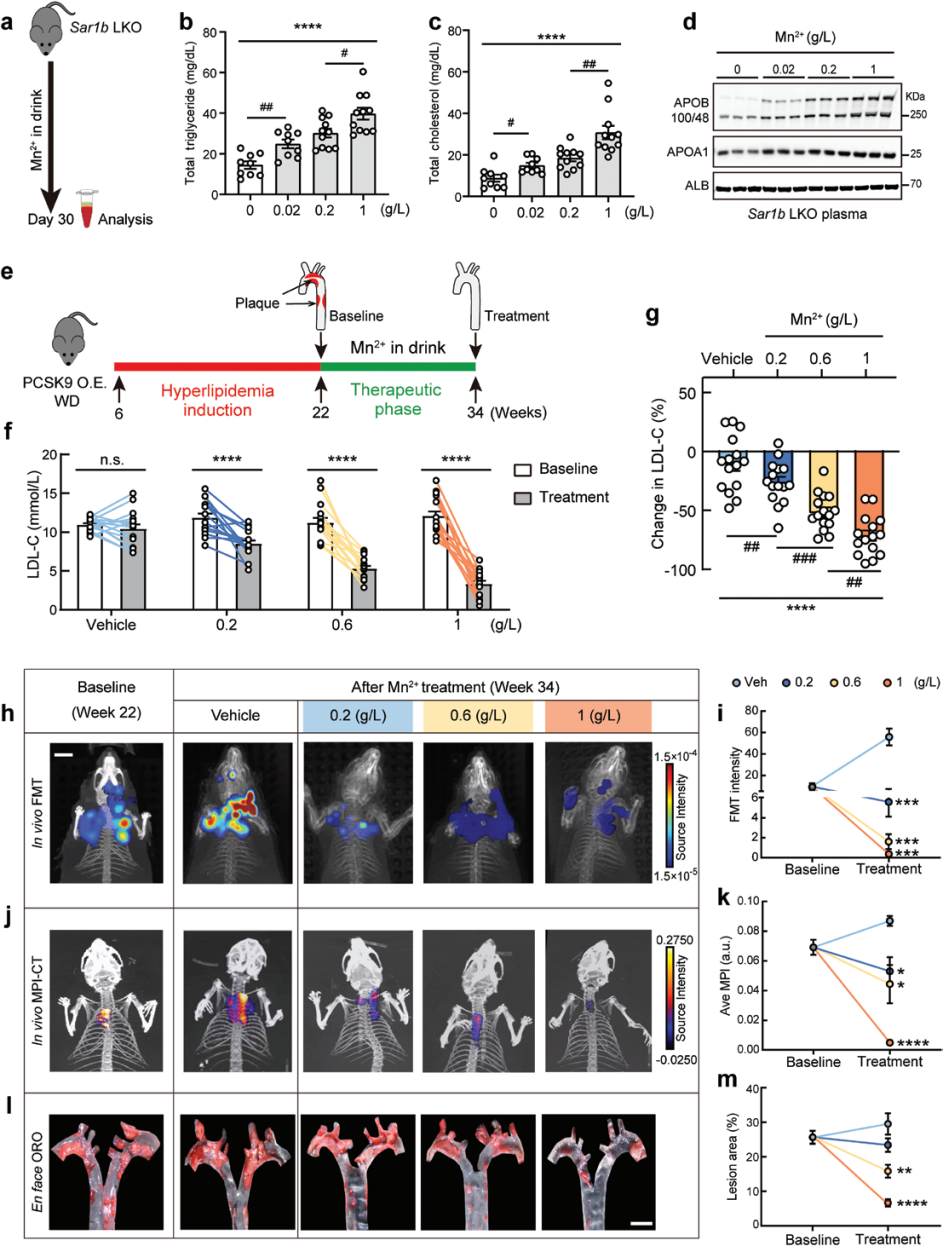
Dietary manganese titration treats dyslipidemia and enables plaque reversal. (a) Experimental design of treating hypolipidemia in *Sar1b* LKO mice with Mn^2+^ supplemented in the drink. (b) Mn^2+^ supplement restores plasma triglyceride levels in a dose-dependent manner in *Sar1b* LKO mice. *n* = 9, 9, 11, 11 mice for 0, 0.02, 0.2, 1 g/L, respectively. Data are presented as mean ± SEM. ^****^P < 0.0001 by one-way ANOVA test. ^#^*P* < 0.05, ^##^*P *< 0.01 by the posthoc test of Tukey. (c) Mn^2+^ supplement restores plasma cholesterol level in a dose-dependent manner in *Sar1b* LKO mice. *n* = 9, 9, 11, 11 mice for 0, 0.02, 0.2, 1 g/L, respectively. Data are presented as mean ± SEM. ^****^P < 0.0001 by one-way ANOVA test. ^#^*P* < 0.05, ^##^*P *< 0.01 by the posthoc test of Tukey. (d) Immunoblotting analysis of plasma samples from Mn^2+^-supplemented *Sar1b* LKO mice in (a). Representative of three independent experiments is shown. (e) Experimental design of treating atherosclerosis with Mn^2+^ supplemented in the drink. (f) Plasma LDL-C levels of mice with pathogenic ­induction before (baseline) and after Mn^2+^ treatment. *n* = 15 mice for each group. Data are presented as mean ± SEM. ^****^P < 0.0001 by paired Student’s *t*-test. n.s., not significant. (g) Percentage changes in LDL-C levels from baseline by Mn^2+^ treatment in (e). *n* = 15 mice for each group. Data are presented as mean ± SEM. ^****^P < 0.0001 by one-way ANOVA test. ^##^*P* < 0.01, ^###^*P* < 0.001 by the posthoc test of Tukey. (h) Representative of *in vivo* FMT imaging of 5HFeC NPs in aortae from mice at baseline and after vehicle or Mn^2+^ treatment (Color: fluorescence, gray scale: CT). (i) Reversal curves of unstable atherosclerotic plaques revealed by 5HFeC NP fluorescence signal, as a response to Mn^2+^ treatments. *n* = 5 mice for baseline and each treatment. Data are presented as mean ± SEM. ^***^P < 0.001 by unpaired two-sided Student’s *t*-test. (j) Representative of *in vivo* 3D-MPI-CT imaging of 5HFeC NPs in aortae from mice at baseline and after vehicle or Mn^2+^ treatment (Color: MPI, gray scale: CT). (k) Reversal curves of unstable atherosclerotic plaques revealed by 5HFeC NP MPI signal, as a response to Mn^2+^ treatments. *n* = 3 mice for baseline and each treatment. Data are presented as mean ± SEM. ^*^P < 0.05, ^****^P < 0.0001 by unpaired two-sided Student’s *t*-test. (l) Representative images of *en face* Oil Red O (ORO) staining of the aorta from mice at baseline and after vehicle or Mn^2+^ treatment. (m) Atherosclerosis reversal curves as a response to Mn^2+^ treatments. *n* = 9 mice for baseline and each treatment. Data are presented as mean ± SEM. ^**^P < 0.01, ^****^P < 0.0001 by unpaired two-sided Student’s *t*-test.

The above results, together with the previously reported ­*tunable* manganese signal in COPII condensation and subsequent lipid delivery [10], led us to hypothesize a potential manganese therapy for broad-spectrum lipid disorders. To test this hypothesis, we first employed hepatic *Sar1b*-deficient mice (*Sar1b* LKO), which exhibited hypolipidemia due to impaired COPII function and consequently led to defective lipoprotein secretion. We devised a treatment regimen involving varying doses of Mn^2+^ supplementation through drinking water to quantitatively scrutinize the potential manganese therapy (Fig. 1a), with the hypothesis that boosting the Mn^2+^ signal would rescue the hypolipidemia caused by crippled COPII function.

Consistent with the above, the diminished lipid levels in *Sar1b* LKO mice, including triglycerides and cholesterol, were significantly elevated when Mn^2+^ was supplemented in the drink for only 4 weeks (Fig. 1b and c). Consistent changes were also observed in the plasma fast protein liquid chromatography (FPLC) profiles (Supplementary Fig. S4a and b). Importantly, the restorative impact on plasma lipids demonstrated a direct correlation with the administered manganese dosages, underscoring the tunable quality of the condensation-regulating manganese signal [10]. Consistent with a rescue of defects in lipoprotein transport, manganese supplementation also dose-dependently elevated the initially depleted levels of plasma APOB in *Sar1b* LKO mice, while plasma albumin remained unaltered (Fig. 1d). Tissues including heart, brain, gut, and muscle appeared grossly normal (Supplementary Fig. S4c). Taken together, the data from genetic models of *Sar1b* LKO uncovered a specific and tunable effect of manganese signal in treating hypolipidemia, representing one end of the spectrum of lipid disorders.

The above results led us to further examine the potential of manganese therapy for treating the common hyperlipidemia, and whether such therapeutic effects could even turn into the reversal of atherosclerotic plaques. To this end, we designed a treatment strategy (Fig. 1e) on an established pathological model with ectopic proprotein convertase subtilisin/kexin type 9 (PCSK9) expression and Western diet feeding, which effectively induced hyperlipidemia and atherosclerosis. To precisely control manganese levels during the pathogenic phase, we customized a Mn-deficient Western diet and supplied 0.02 g/L Mn^2+^ in drinking water. This level of Mn^2+^ supply mimics the normal dietary manganese supply. After confirming the induction of pathogenic hyperlipidemia and atherosclerosis, these mice would be subjected to the therapeutic phase. Specifically, mice were randomly assigned to the control vehicle group (control), or one of the three treatment groups that received a therapeutic dose of Mn^2+^ via drinking water at doses of 0.2, 0.6, or 1 g/L, based on our previous titration [10].

After 16 weeks of pathogenic induction, the mice developed hyperlipidemia as expected and were then subjected to manganese treatment for another 12 weeks. A 1.53% increase in plasma cholesterol was observed in the vehicle control group compared to the baseline at the initiation of the treatment phase, further supporting the successful establishment of the pathogenic model. Of note, Mn^2+^ treatment led to dose-dependent reductions in the atherogenic LDL-C from the baseline in the groups, with a 28.27% reduction by 0.2 g/L Mn^2+^, a 53.07% reduction by 0.6 g/L Mn^2+^, and a 72.84% reduction by 1 g/L Mn^2+^ (Fig. 1f and g). Similar dose-dependent reductions were also observed in total circulating cholesterol (Supplementary Fig. S5a and b) and triglyceride (Supplementary Fig. S5c and d). Profiling of plasma lipids by FPLC further confirmed the reduction of atherogenic lipoproteins including VLDL and LDL (Supplementary Fig. S5e and f). Accordingly, Mn^2+^ treatment decreased plasma APOB levels in a dose-dependent manner (Supplementary Fig. S5g). Of note, plasma ALT and AST levels, while slightly elevated by the atherogenic diets, were even reduced in the Mn^2+^ treatment groups compared to the controls (Supplementary Fig. S5h). Plasma creatine kinase remained unchanged in all groups (Supplementary Fig. S5i). In conclusion, the results demonstrated that Mn^2+^ treatment enabled the quantitative reduction of the pro-atherogenic lipids, without inducing apparent liver damage.

The intensive lipid-lowering effects of the manganese therapy led us to further examine its therapeutic potential in the reversal of atherosclerotic plaques, a goal that has yet to be achieved even in pre-clinical models. We utilized the afore-described murine models of hyperlipidemia as the primary driver of athero­sclerosis and related CVDs. To quantitatively track the progression or reversal of atherosclerosis in these hyperlipidemic mice, we employed the recently developed 5-HT-Fe_3_O_4_-Cy7 nanoparticle (5HFeC NPs) as the dual-modal *in vivo* imaging probe. These NPs preferentially target macrophage-derived myeloperoxidases in vulnerable and unstable plaques and the signal could be detected *in vivo* with both fluorescence molecular tomography (FMT) and 3D magnetic particle imaging (3D-MPI) [12]. We further calibrated the *in vivo* dual-mode imaging with *en face* analysis of Oil Red O samples that are routinely performed to assess atherosclerosis (Supplementary Fig. S6a and b). Both FMT (fluorescent) and 3D-MPI (magnetic) signals of 5HFeC NPs significantly elevated in the aortae of the mice after 16 weeks of pathogenic induction. *En face*, Oil Red O staining also confirmed extensive atherosclerotic plaques in these mice (Supplementary Fig. S6c).

Of note, after a 12-week treatment period, the 5HFeC NP fluorescence signal decreased substantially in live mice, in a manner corresponding to the Mn^2+^ doses (Fig. 1h and i). When compared to the baseline upon initiation of the Mn^2+^ treatment, all treated groups exhibited Mn^2+^-dose-dependent reduction in FMT signal, suggesting effective reversal of atherosclerotic plaques. Strikingly, in mice receiving the highest therapeutic doses (1 g/L), the 5HFeC NP signals became nearly absent in the aortae. Consistently, 3D-MPI imaging and quantification further confirmed the successful reversal of existing and likely unstable atherosclerotic plaques by the Mn^2+^ therapy (Fig. 1j and k).

*En face* staining of the aorta further confirmed a similar ­dose-dependent plaque reversal responding to Mn^2+^ treatment (Fig. 1l and m). When compared to the baseline, treatment with 0.2 g/L Mn^2+^ caused an 8.71% reduction, 0.6 g/L Mn^2+^ caused a 38.27% reduction, and 1 g/L Mn^2+^ caused a 75.63% reduction in atherosclerotic plaques, in sharp contrast to a 15.16% increase in the vehicle group. Consistently, the *ex vivo* analysis also confirmed that Mn^2+^ treatment at the 1 g/L dose enabled a substantial reversal of atherosclerotic plaques developed during the 4-month pathogenic phases, and the remaining Oil Red O signal may reflect smaller and rather stable plaques. Taken together, these data in murine disease models demonstrated the feasibility of manganese-based therapy to achieve plaque reversal via intensive lipid lowering.

Taken together with the recent mechanistic study [10], data presented in the current study further demonstrated that Mn^2+^, as an endogenous messenger promoting COPII condensation, selectively regulates lipoprotein transport and systemic lipid homeostasis. The novel function of the manganese messenger could be harnessed for therapeutic interventions targeting lipid disorders, paving the way for novel treatments for cardiometabolic diseases. Moreover, the unique bell-shaped regulation by manganese dosage could enable tailored treatment options based on therapeutic needs, further highlighting the versatility and potentially broad application of the novel strategy.

Manganese is an essential trace element crucial for health [13]. In humans and mice, manganese is primarily absorbed by intestinal enterocytes through different transporters en route to various tissues via the bloodstream, with the liver acting as the main manganese reservoir [11]. While being an essential trace element in biology, excess manganese also exerts neuro-toxicity over time, especially when inhaled in miners. Moreover, mutations in Mn^2+^ transporters including SLC39A14 and SLC30A10 in humans also cause manganese overload. Nevertheless, consumption of manganese-rich diets containing up to 2000 ppm manganese for months in mice (corresponding to 2 g/L in drinking water in our study) did not appear to cause obvious toxicity. These previous data are consistent with the lack of overt toxicity in our study with the manganese therapy for 3 months, though more extensive, future neurological analysis may be required to further substantiate the safety of manganese supplementation. Moreover, despite the striking therapeutic effects for treating dyslipidemia and reversing atherosclerotic plaques in the current study, the efficiency of dietary manganese absorption is rather low in both mice and humans. Hence, the means to harness the benefits while limiting the potential harms of the manganese signal warrant future elucidation. One could envision that dosage control, tissue-specific targeting, and/or mobilizing endogenous manganese stores within liver cells could be more precise strategies with likely greater efficacy.

While the canonical function of manganese mostly concerns its passive, required roles as enzymatic co-factors [11], our recent studies uncovered an active, signal-based role of the small mole­­cule in promoting COPII condensation, which prioritizes the transport of lipoproteins. The anti-atherosclerotic effects of manganese have been observed in cholesterol-fed ­rabbits with little mechanistic explanation [14], whereas manganese admini­stration prevented atherosclerosis in mice by targeting endothelial cells without affecting lipid profiles [15]. While these and our studies highlight the multifaceted cardiometabolic benefits of manganese administration, regulation of manganese at the molecular level remains poorly understood, especially in the intricate context of physiological and pathological conditions. Moreover, manganese treatment did not induce additional and even alleviated lipid accumulation in the liver of the dyslipidemia mice (according to our unpublished observation), with yet-to-be-elucidated mechanisms. At the organism level, strikingly, even formal guidelines for manganese uptake and toxicity are lacking. Given the potential broad impact on CVDs and metabolic health, future studies on the under-studied essential element could be of high value, including investigations that could be conducted with relevance to humans.

## Supplementary Material

load040_suppl_Supplementary_Material

## Data Availability

The online version of this article contains supplementary material, which is available to authorized users.
