# Supplementary material for: Manganese therapy for dyslipidemia and plaque reversal in murine models
Source: Life Metab. 2023 Oct 26;2(6):load040. doi: 10.1093/lifemeta/load040 (PMC11749556; doi:10.1093/lifemeta/load040)
Supplement: load040_suppl_Supplementary_Material [file load040_suppl_Supplementary_Material.pdf]

Supplementary Materials for

Manganese Therapy for Dyslipidemia and Plaque Reversal in Murine Models

Supplementary Methods

Mouse models

All animal housing and use were approved by the Institutional Animal Care and Use Committees of Peking University, an AAALAC accredited laboratory animal facility. Mice were housed under standardized conditions, including a temperature of 22˚C, a 12-hour light/dark cycle, and a humidity level of 40%–60%. Mice had free access to food and water, unless otherwise specified. Male mice were used in all experiments. Mice were randomly assigned to different experimental groups.

For treating hypolipidemia experiments, *Sar1b*$^{fl/fl}$ mice (KOMP: 061774, RRID: MMRRC_061774-UCD) were injected with adeno-associated virus (AAV) containing thyroxine-binding globulin (TBG) driven Cre recombinase (AAV-TBG-Cre) to generate *Sar1b* liver-specific knockout mice (*Sar1b* LKO). *Sar1b* LKO mice were fed ad lib with AIN-76A diet with no added manganese, and have free access to water supplemented with Mn^{2+} at corresponding doses as indicated.

For murine models of hyperlipidemia and atherosclerosis, 6-week-old wild type mice receiving AAV-hPCSK9^{D374Y} were fed ad lib with manganese deficient Western diets (MolDiets, M20120701) and water containing 0.02 g/L Mn^{2+} for 16 weeks to induce hyperlipidemia and atherosclerotic lesions. Then they were randomly allocated to 4 groups with extra Mn^{2+} supplemented in drink as indicated for another 12 weeks.

Determination of Mn LD_{50}

To determine the LD_{50} of Mn, a single oral gavage was given to mice with 0, 0.05, 0.1, 0.15, 0.2, 0.25, 0.3, 0.4, 0.5 or 1 g/kg $MnCl_2$ in 0.2 mL H_2O (n=4 mice per dose). The survival of the mice was monitored for 1 week. Mice received with doses of 0.4, 0.5, and 1 g/kg exhibited paralysis within minutes and subsequently died. No signs of paralysis or lethality were observed at any time with doses equal to or lower than 0.25 g/kg.

Plasma characterization and fast-protein liquid chromatography (FPLC) analysis

Blood samples were collected from tail tips of 16-h fasted mice via heparin capillary. Plasma was obtained by centrifugation at 6000 rpm, 4°C for 10 min. Triglyceride, total cholesterol, LDL-Cholesterol, ALT/GPT, AST/GOT, creatine kinase, and ketone levels were measured using respective commercial kits (TR0100 of Sigma; 000180 of Zhongsheng beikong; A113-1-1 of Nanjing Jiancheng Bioengineering Institute; 000000010, 000000020, N/A of Zhongsheng beikong; BC5060 of Solarbio, respectively) according to the manufacturer's protocol. For FPLC analysis, pooled plasma samples of the same treatment were fractionated using Superose 6 columns. Fractions were collected at a flow rate of 0.5 mL/min for subsequent cholesterol and triglyceride measurements.

Adeno-Associated Virus (AAV) vector and viral delivery

AAV packaging and purification were carried out as previously described (ref). AAV were delivered via tail vein injection. To obtain *Sar1b* LKO mice, each *Sar1b*$^{fl/fl}$ mouse at 6-week-old was simultaneously injected with 1E^{11} of AAV-TBG-Cre viral particles. For murine models of hyperlipidemia, each wild type mice were received 2E^{10} of hPCSK9^{D374Y} viral particles

RNA extraction and RNA-seq analysis

Total RNA was extracted from frozen liver samples using Trizol reagent following the manufacturer's instructions. mRNA-seq analysis was performed by Novogene, Beijing, using the Illumina Novaseq 6000 platform. The following data analysis pipeline was employed for total liver mRNA-seq. Paired-end reads in the Fastq format with a length of 150 bp were downloaded, and FastQC was employed for pre-mapping sequencing quality assessment. The reads were then mapped to the mouse genome (mm10) using HISAT2 with default parameters, specifically selecting uniquely mapped reads. The raw gene counts for each sample using htseq-count from HTSeq and Fragments Per Kb of exon per Million mapped reads (FPKM) were calculated using StringTie. Differential gene expression analysis was performed using DESeq2 and the differential expressed genes with fold change greater than 2 and FDR less than 0.05 were visualized as volcano plot by ggplot2. The Gene Set Enrichment Analysis (GSEA) was performed on the ranked data sets according to $\log_2$(Foldchange) by clusterProfiler using the Gene Ontology (GO) database.

Metal measurement using ICP-MS

The liver samples from mice were subjected to acid digestion using 100% HNO_3 at 140°C for 30 min. The microelement content was then measured using Inductively Coupled Plasma Mass Spectrometry (ICP-MS) on a PerkinElmer NexION 350X instrument. To measure metal concentrations in the plasma, plasma samples were diluted with a diluent containing 4% isopropanol, 1% NH_3/H_2O, 0.01% EDTA, and 0.01% Triton X-100. The diluted samples were analyzed using ICP-MS/MS on an Agilent 8900 instrument. Calibration was performed using the internal standard 45Sc and a serial dilution of an external standard mix prepared from Agilent 5183-4688.

Immunoblotting

Liver samples were lysed using ice-cold lysis buffer containing 50 mM Tris-Cl (pH 7.4), 137 mM NaCl, 1 mM EGTA, 5 mM $MgCl_2$, 1% Nonidet P-40, and 10% glycerol, supplemented with protease inhibitor tablets (Roche), followed by centrifugation at 13,000 rpm for 10 min to obtain solubilized lysates. Proteins were resolved by 3 to 15% Tris-acetate SDS-PAGE and transferred onto nitrocellulose membranes, followed by analyzed by indicated antibodies.

Histology

Tissue samples were collected and fixed in 4% PFA. Tissue embedding, sectioning, and hematoxylin and eosin (H&E) staining were performed Pathology Center at Peking

University or Beijing ZKWB-Bio Biotechnology Co., Ltd. For Oil Red O staining, tissues were embedded in OCT compound and snap-frozen. Cryo-sections with a thickness of 8 μm were obtained and stained with Oil Red O as per the manufacturer's instructions. Immunohistochemistry was performed on paraffin-embedded liver sections. Deparaffinization and rehydration were followed by antigen retrieval. The sections were blocked with 10% goat serum for 30 min and incubated overnight at 4°C with primary antibodies diluted in blocking buffer. Subsequently, the sections were incubated with secondary antibodies for 2 h at room temperature. Finally, visualization was achieved by using DAB (3,3'-diaminobenzidine) staining.

Quantification of hepatic triglycerides and cholesterol

Liver samples were weighed and then homogenized in PBS. Lipids were extracted from the homogenates following established protocols of modified Bligh-Dyer method. In summary, the homogenates were vigorously mixed with a chloroform-methanol mixture (2:1). After centrifugation, the organic phase was carefully collected and concentrated using a rotary evaporator under vacuum. The resulting lipid extract was reconstituted in a solution of 15% Triton X-100 in ddH$_2$O. The quantification of triglycerides and cholesterol was performed as previously outlined.

***in vivo* and *en face* analysis of atherosclerosis**

12 hours prior to *in vivo* imaging of atherosclerosis, mice were intravenously injected with 5HFeC NPs (7.5 mg Fe per kg weight), which were synthesized according to previously reported procedure. Fluorescence molecular tomographic imaging was performed using the IVIS spectrum (PerkinElmer, USA), and quantitative analysis was done using Living Image Software (Caliper Life Sciences). 3D Magnetic Particle Imaging (MPI) imaging was performed using an MPI scanner (MOMENTUM, Magnetic Insight, Inc., Alameda, CA, USA) with specific parameters. CT imaging was performed with an in-house-built CT scanner. The MPI-CT images were reconstructed, co-registered and analyzed using VivoQuant software. The 5HFeC NPs with active myeloperoxidase (MPO) targeting ability and related imaging equipment were obtained from CAS Key Laboratory of Molecular Imaging, Institute of Automation, Chinese Academy of Sciences.

For *en face* analysis of atherosclerosis, the isolated aortas were stained with Oil red O and photographed using an Olympus stereo microscope equipped with an SCMOS camera. The lesion areas in the thoracic aorta were quantified using Image J.

Quantification and statistical analysis

All experimental data are presented as mean ± SEM, unless otherwise specified in the figure legends. Sample sizes were not predetermined using statistical methods. Statistical analysis was performed using GraphPad Prism 9. Student's t-test or one-way ANOVA with Tukey's post-hoc test was used for calculating statistical significance, as specified in the figure legends. Results were considered significant when $P < 0.05$. Statistical significance levels were indicated in the figure legends. The presented

experimental results are representative of at least 3 independent experiments. Mouse experiments were conducted with random allocation. Imaging and histology were reviewed in a blinded fashion.

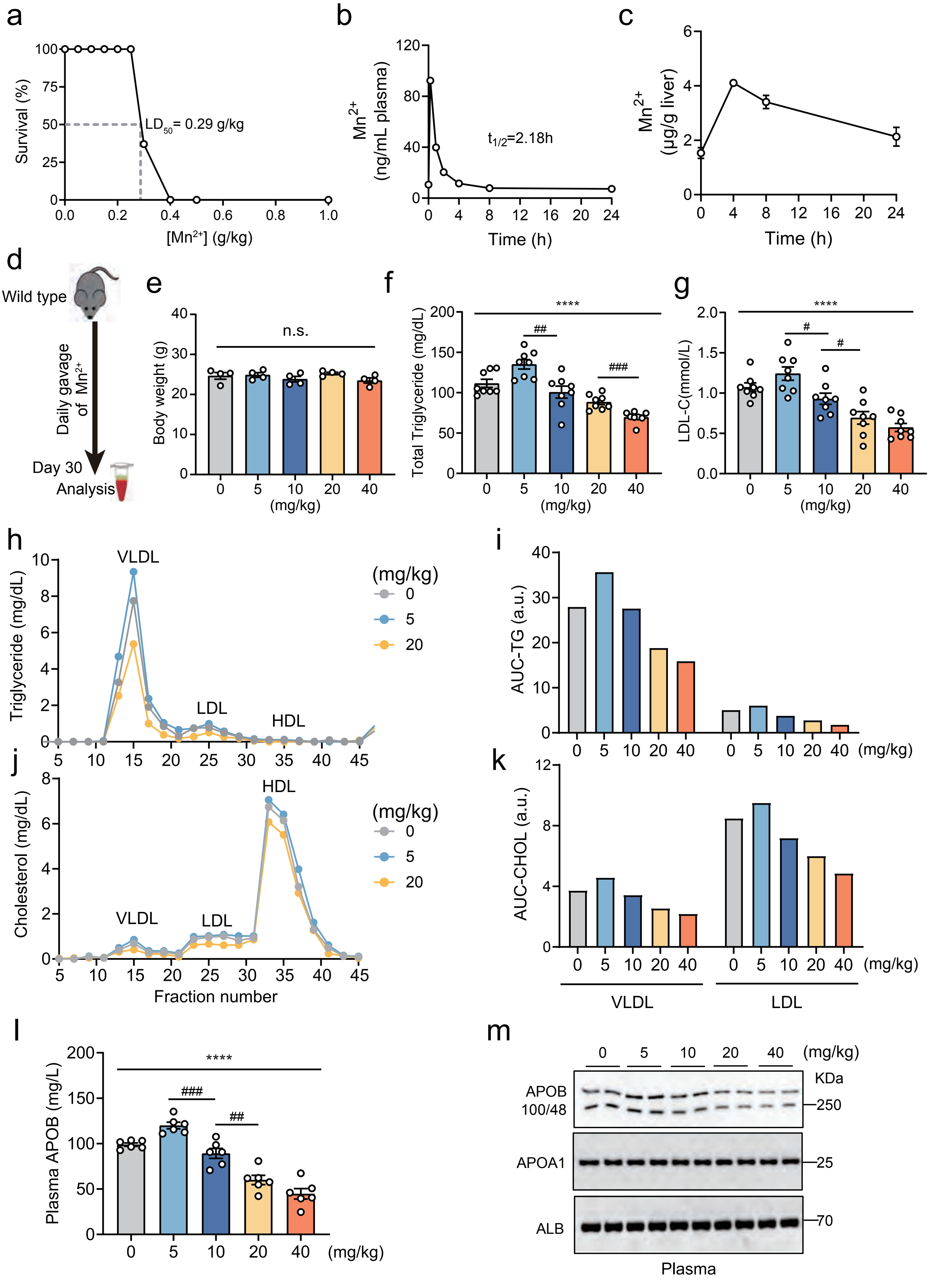
a
Survival (%)
100
75
50
25
0
0.0 0.2 0.4 0.6 0.8 1.0
LD_{50}= 0.29 g/kg
[Mn^{2+}] (g/kg)
b
Mn^{2+} (ng/mL plasma)
120
80
40
0
0 4 8 12 16 20 24
$t_{1/2}$=2.18h
Time (h)
c
Mn^{2+} (μg/g liver)
6
4
2
0
0 4 8 12 16 20 24
Time (h)
d
Wild type
Daily gavage of Mn^{2+}
Day 30
Analysis
e
Body weight (g)
40
30
20
10
0
n.s.
0 5 10 20 40
(mg/kg)
f
Total Triglyceride (mg/dL)
200
150
100
50
0
****
##
###
0 5 10 20 40
(mg/kg)
g
LDL-C(mmol/L)
2.0
1.5
1.0
0.5
0.0
****
#
#
0 5 10 20 40
(mg/kg)
h
Triglyceride (mg/dL)
10
8
6
4
2
0
VLDL
LDL
HDL
5 10 15 20 25 30 35 40 45
(mg/kg)
0
5
20
i
AUC-TG (a.u.)
40
30
20
10
0
0 5 10 20 40 0 5 10 20 40 (mg/kg)
j
Cholesterol (mg/dL)
8
6
4
2
0
VLDL
LDL
HDL
5 10 15 20 25 30 35 40 45
Fraction number
(mg/kg)
0
5
20
k
AUC-CHOL (a.u.)
12
8
4
0
0 5 10 20 40 0 5 10 20 40 (mg/kg)
VLDL
LDL
l
Plasma APOB (mg/L)
200
150
100
50
0
****
###
##
0 5 10 20 40 (mg/kg)
m
0 5 10 20 40 (mg/kg)
KDa
APOB 100/48
250
APOA1
25
ALB
70
Plasma

Figure S1

Figure S1. Manganese regulates plasma lipid levels in wild type adult mice

(a) Fraction of mice surviving after intragastric (i.g.) administration of Mn^{2+} at the indicated dose. n=4 mice for each group.

(b) Plasma Mn^{2+} concentration-time curve following i.g. administration of 30 mg/kg body weight to mice. n=2 mice for each group.

(c) Hepatic Mn^{2+} concentration-time curve following i.g. administration of 30 mg/kg body weight to mice. n=2 mice for each group.

(d) Experimental design of daily gavage of Mn^{2+} in wild type mice on normal chow.

(e) Body weights of the mice with indicated doses of Mn^{2+} daily intake. n=4 mice for each group. Data are presented as mean ± SEM. n.s.: not significant.

(f) Bell-shape regulation of plasma triglyceride levels by oral Mn^{2+} supply. n=8 mice for each group. Data are presented as mean ± SEM. ****: $P < 0.0001$ by one-way ANOVA test. ##: $P < 0.01$; ###: $P < 0.001$ by the posthoc test of Tukey. X-axis: manganese doses (mg/kg/day).

(g) Bell-shape regulation of plasma LDL-cholesterol levels by oral Mn^{2+} supply. n=8 mice for each group. Data are presented as mean ± SEM. ****: $P < 0.0001$ by one-way ANOVA test. #: $P < 0.05$ by the posthoc test of Tukey. X-axis: manganese doses (mg/kg/day).

(h) Pooled plasma samples in (d) were fractionated into VLDL, LDL and HDL by FPLC, followed by triglyceride measurement. Results of mice receiving 0, 5, 20 mg/kg Mn^{2+} were presented.

(i) Quantification of APOB-containing VLDL and LDL triglyceride levels acquired by FPLC as in (h).

(j) Pooled plasma samples in (d) were fractionated as in (h), followed by cholesterol measurement. Results of mice receiving 0, 5, 20 mg/kg Mn^{2+} were presented.

(k) Quantification of APOB-containing VLDL and LDL cholesterol levels acquired by FPLC as in (j)

(l) Bell-shape regulation of plasma APOB levels by oral Mn^{2+} supply. n=6 mice for each group. Data are presented as mean ± SEM. ****: $P < 0.0001$ by one-way ANOVA test. ##: $P < 0.01$; ###: $P < 0.001$ by the posthoc test of Tukey. X-axis: manganese doses (mg/kg/day).

(m) IB analysis of plasma samples from mice with indicated doses of Mn^{2+} daily intake. Representative of three independent experiments is shown.

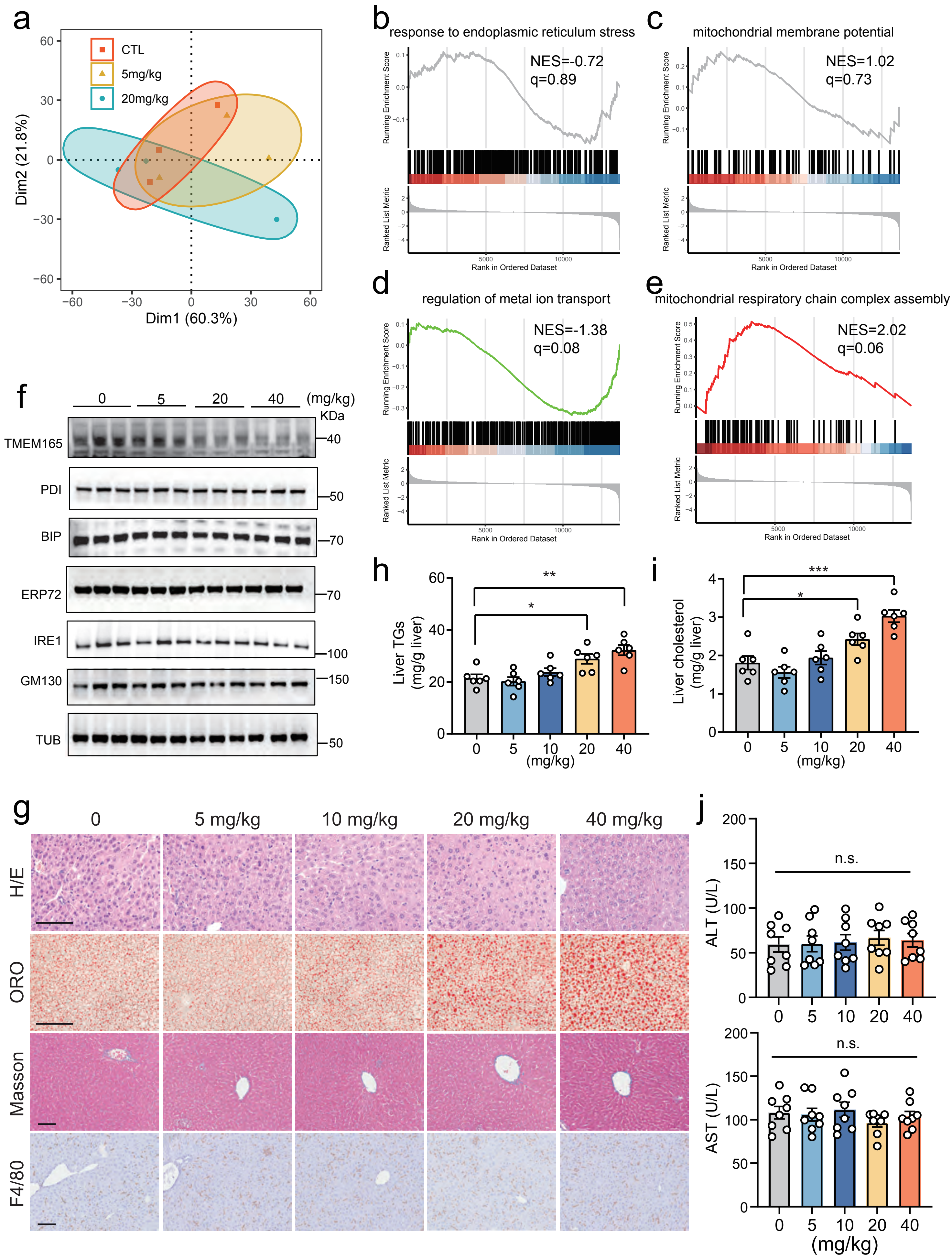

a
CTL
5mg/kg
20mg/kg
Dim1 (60.3%)
Dim2 (21.8%)
b
response to endoplasmic reticulum stress
NES=-0.72
q=0.89
c
mitochondrial membrane potential
NES=1.02
q=0.73
d
regulation of metal ion transport
NES=-1.38
q=0.08
e
mitochondrial respiratory chain complex assembly
NES=2.02
q=0.06
Running Enrichment Score
Ranked List Metric
Rank in Ordered Dataset
f
0
5
20
40
(mg/kg)
KDa
TMEM165
PDI
BIP
ERP72
IRE1
GM130
TUB
h
Liver TGs (mg/g liver)
(mg/kg)
i
Liver cholesterol (mg/g liver)
g
0
5 mg/kg
10 mg/kg
20 mg/kg
40 mg/kg
H/E
ORO
Masson
F4/80
j
ALT (U/L)
AST (U/L)
n.s.
(mg/kg)

Figure S2

Figure S2. Manganese enables safe and selective regulation of blood lipid secretion

(a) Principle component analysis of mRNA profiles in the liver samples from mice with Mn^{2+} administration at indicated doses. n=3 for each group.

(b-e) GSEA enrichment analysis of mRNA expression profiles in the liver sample from vehicle control and the 20mg/kg group.

(f) IB of liver samples from mice with indicated doses of Mn^{2+} supply. Representative of three independent experiments is shown.

(g) Histology on liver samples from vehicle control mice or mice receiving Mn^{2+} gavage at indicated doses. Scale bar=100μm

(h) Hepatic TG in the mice with indicated doses of Mn^{2+} daily intake. n=6 mice for each group. Data are presented as mean ± SEM. *: $P < 0.05$; **: $P < 0.01$ by one-way ANOVA with the posthoc test of Tukey.

(i) Hepatic cholesterol in the mice with indicated doses of Mn^{2+} daily intake. n=6 mice for each group. Data are presented as mean ± SEM. *: $P < 0.05$; ***: $P < 0.001$ by one-way ANOVA with the posthoc test of Tukey.

(j) Plasma ALT and AST levels in control mice or mice receiving Mn^{2+} gavage at indicated doses. n=8 mice for each group. n.s.: not significant.

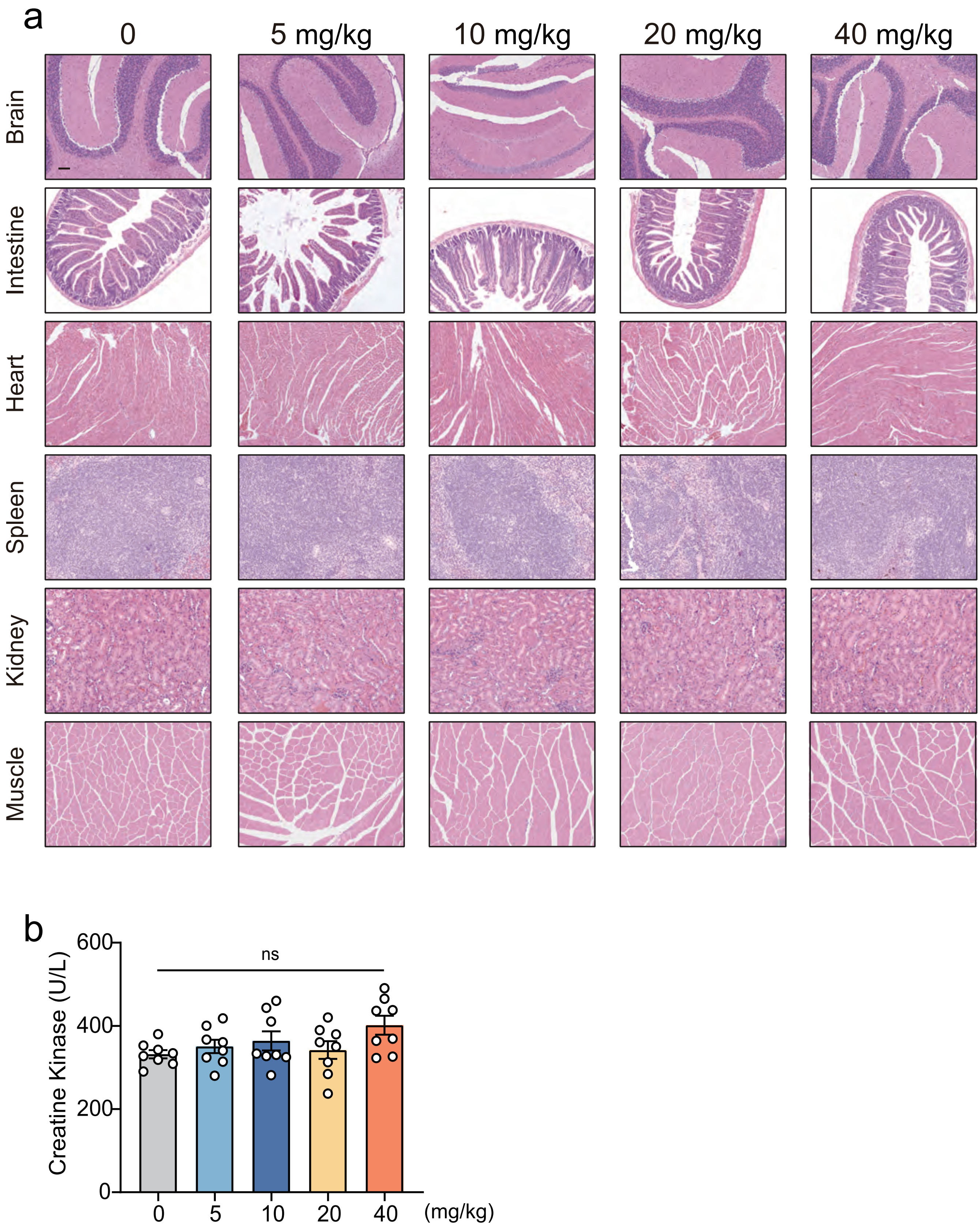
a
0
5 mg/kg
10 mg/kg
20 mg/kg
40 mg/kg
Brain
Intestine
Heart
Spleen
Kidney
Muscle
b
Creatine Kinase (U/L)
600
400
200
0
ns
0
5
10
20
40
(mg/kg)

Figure S3

Figure S3. Overall healthy states in Mn^{2+} administered mice

(a) Histology of extra-hepatic tissues from the mice with indicated doses of Mn^{2+} daily intake. Scale bar=100µm.
(b) Plasma CK levels in control mice or mice receiving Mn^{2+} gavage at indicated doses. n=8 mice for each group. n.s.: not significant.

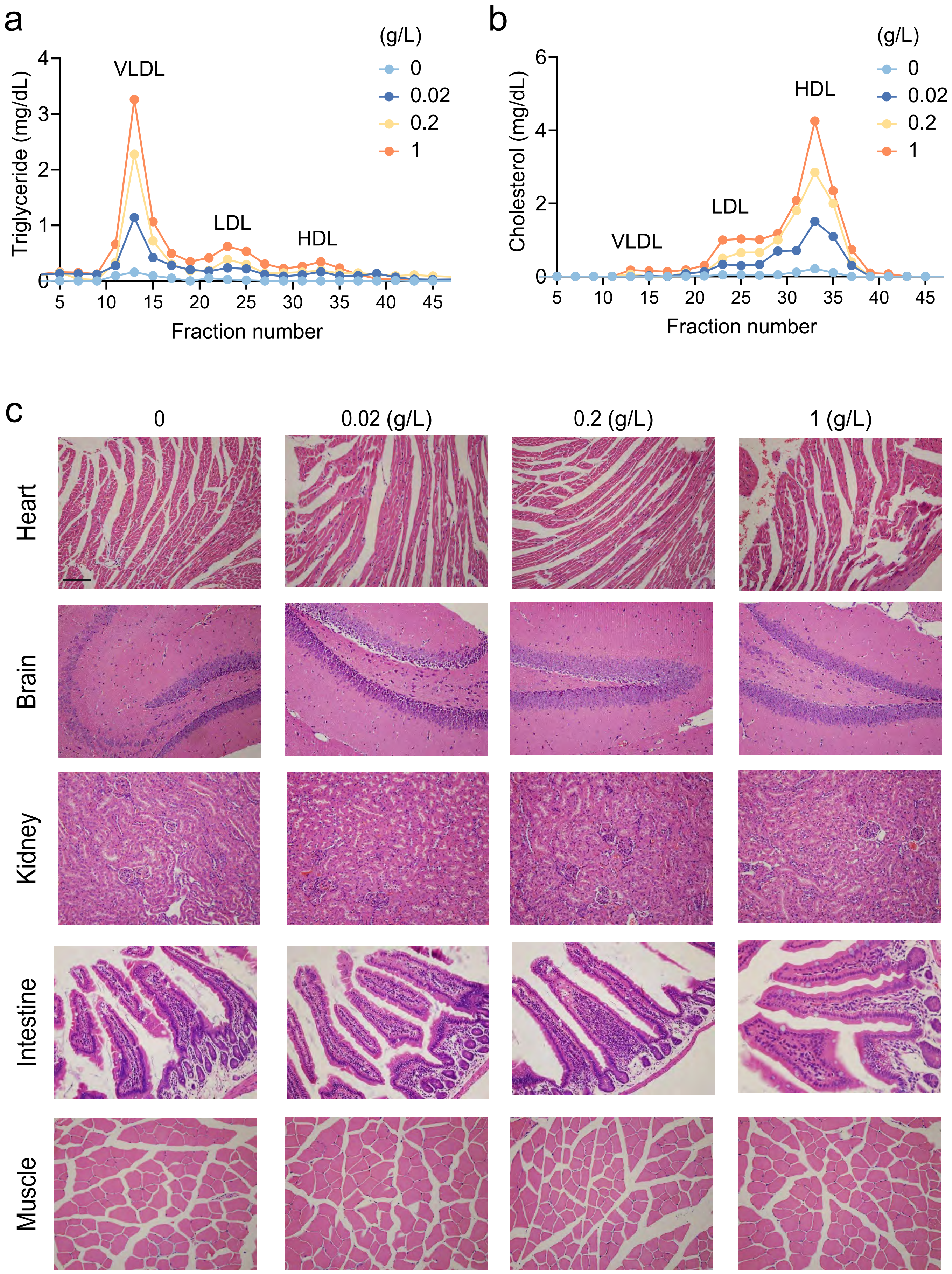
a
Triglyceride (mg/dL)
VLDL
LDL
HDL
Fraction number
(g/L)
0
0.02
0.2
1
b
Cholesterol (mg/dL)
VLDL
LDL
HDL
Fraction number
(g/L)
0
0.02
0.2
1
c
0
0.02 (g/L)
0.2 (g/L)
1 (g/L)
Heart
Brain
Kidney
Intestine
Muscle

Figure S4

Figure S4. Dietary manganese titration treats hypolipidemia in rare lipid disorders

(a) Pooled plasma samples in Figure 1a were fractionated into VLDL, LDL and HDL by FPLC, followed by triglyceride measurement.
(b) Pooled plasma samples in Figure 1a were fractionated as in (a), followed by cholesterol measurement.
(c) Histology of tissues from Mn-supplemented *Sar1b* LKO mice. Scale bar=100μm.

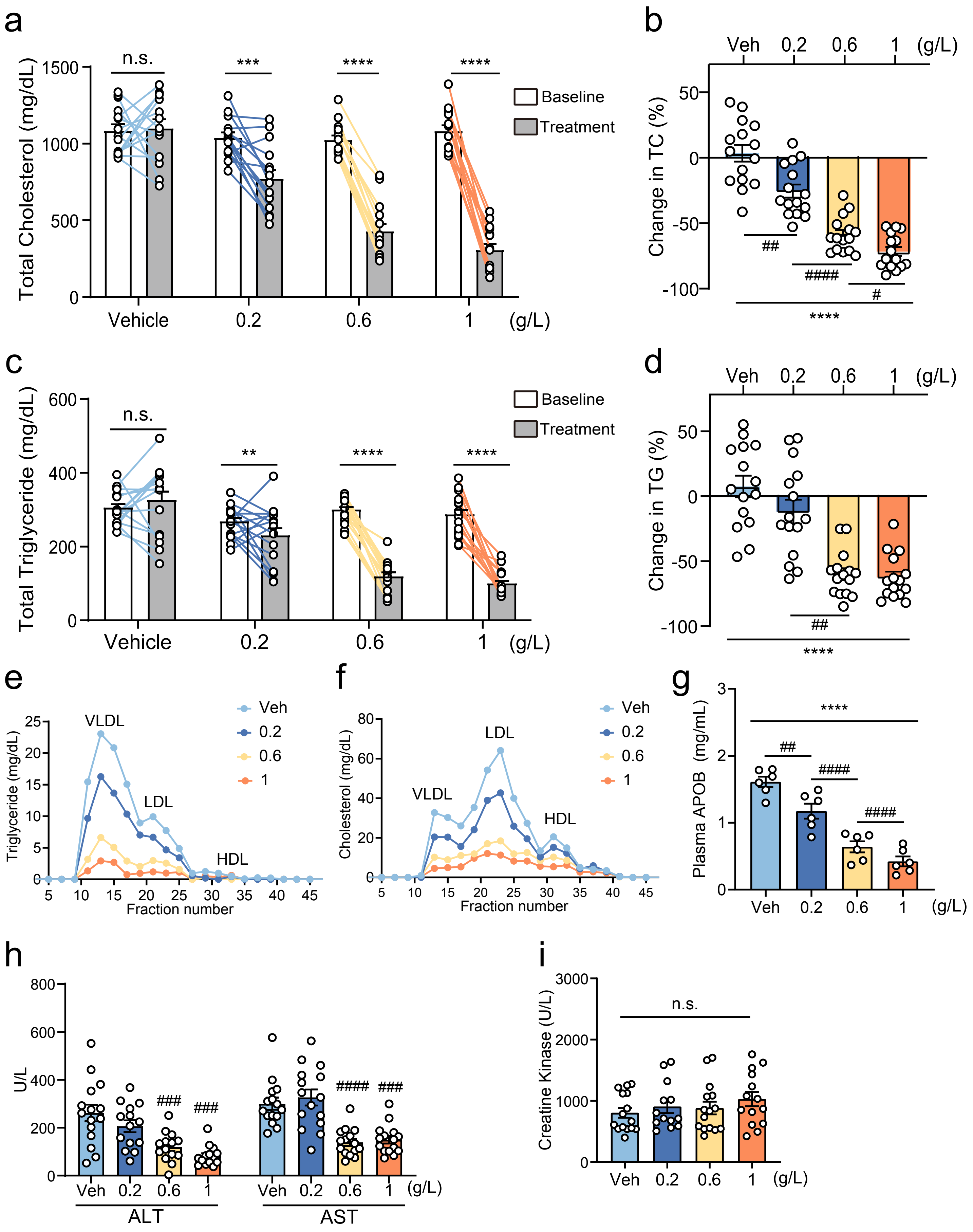

a
Total Cholesterol (mg/dL)
n.s.
***
****
****
Baseline
Treatment
Vehicle
0.2
0.6
1
(g/L)
b
Veh 0.2 0.6 1 (g/L)
Change in TC (%)
##
####
#
****
c
Total Triglyceride (mg/dL)
n.s.
**
****
****
Baseline
Treatment
Vehicle
0.2
0.6
1
(g/L)
d
Veh 0.2 0.6 1 (g/L)
Change in TG (%)
##
****
e
Triglyceride (mg/dL)
VLDL
LDL
HDL
Veh
0.2
0.6
1
Fraction number
f
Cholesterol (mg/dL)
VLDL
LDL
HDL
Veh
0.2
0.6
1
Fraction number
g
Plasma APOB (mg/mL)
****
##
####
####
Veh 0.2 0.6 1 (g/L)
h
U/L
###
###
####
###
Veh 0.2 0.6 1 Veh 0.2 0.6 1 (g/L)
ALT
AST
i
Creatine Kinase (U/L)
n.s.
Veh 0.2 0.6 1 (g/L)

Figure S5

Figure S5. Dietary manganese titration treats pathological hyperlipidemia

(a) Plasma cholesterol levels of mice with pathogenic induction before (baseline) and after Mn^{2+} treatment. n=15 mice for each group. Data are presented as mean ± SEM. ***: $P < 0.001$; ****: $P < 0.0001$ by paired Student's t test. n.s.: not significant.

(b) Percentage changes in plasma cholesterol levels from baseline by Mn^{2+} treatment in (a). n=15 mice for each group. Data are presented as mean ± SEM. ****: $P < 0.0001$ by one-way ANOVA test. #: $P < 0.05$; ##: $P < 0.01$; ###: $P < 0.001$ by the posthoc test of Tukey.

(c) Plasma triglyceride levels of mice with pathogenic induction before (baseline) and after Mn^{2+} treatment. n=15 mice for each group. Data are presented as mean ± SEM. **: $P < 0.01$; ****: $P < 0.0001$ by paired Student's t test. n.s.: not significant.

(d) Changes in plasma triglyceride levels from baseline to the analysis time point after Mn^{2+} treatment in (c). N=15 mice for each group. Data are presented as mean ± SEM. ****: $P < 0.0001$ by one-way ANOVA test. ##: $P < 0.01$ by the posthoc test of Tukey.

(e) Pooled plasma samples of vehicle or Mn^{2+} treated mice were fractionated into VLDL, LDL and HDL by FPLC, followed by triglyceride measurement.

(f) Pooled plasma samples of vehicle or Mn^{2+} treated mice were fractionated as in (e), followed by cholesterol measurement.

(g) Dose dependent reduction in plasma APOB by Mn^{2+} treatment in hyperlipidemic mice. n=6 mice for each group. Data are presented as mean ± SEM. ****: $P < 0.0001$ by one-way ANOVA test. ##: $P < 0.01$; ###: $P < 0.001$ by the posthoc test of Tukey.

(h) Plasma ALT and AST levels of vehicle or Mn^{2+} treated mice. n=15 mice for each group. Data are presented as mean ± SEM. ****: $P < 0.0001$ by one-way ANOVA test. ###: $P < 0.001$; ####: $P < 0.0001$ by the posthoc test of Tukey.

(i) Plasma CK levels in control mice or mice receiving Mn^{2+} gavage at indicated doses. n=15, 13, 15, 14 mice for 0, 0.2, 0.6, 1 g/L, respectively. n.s.: not significant.

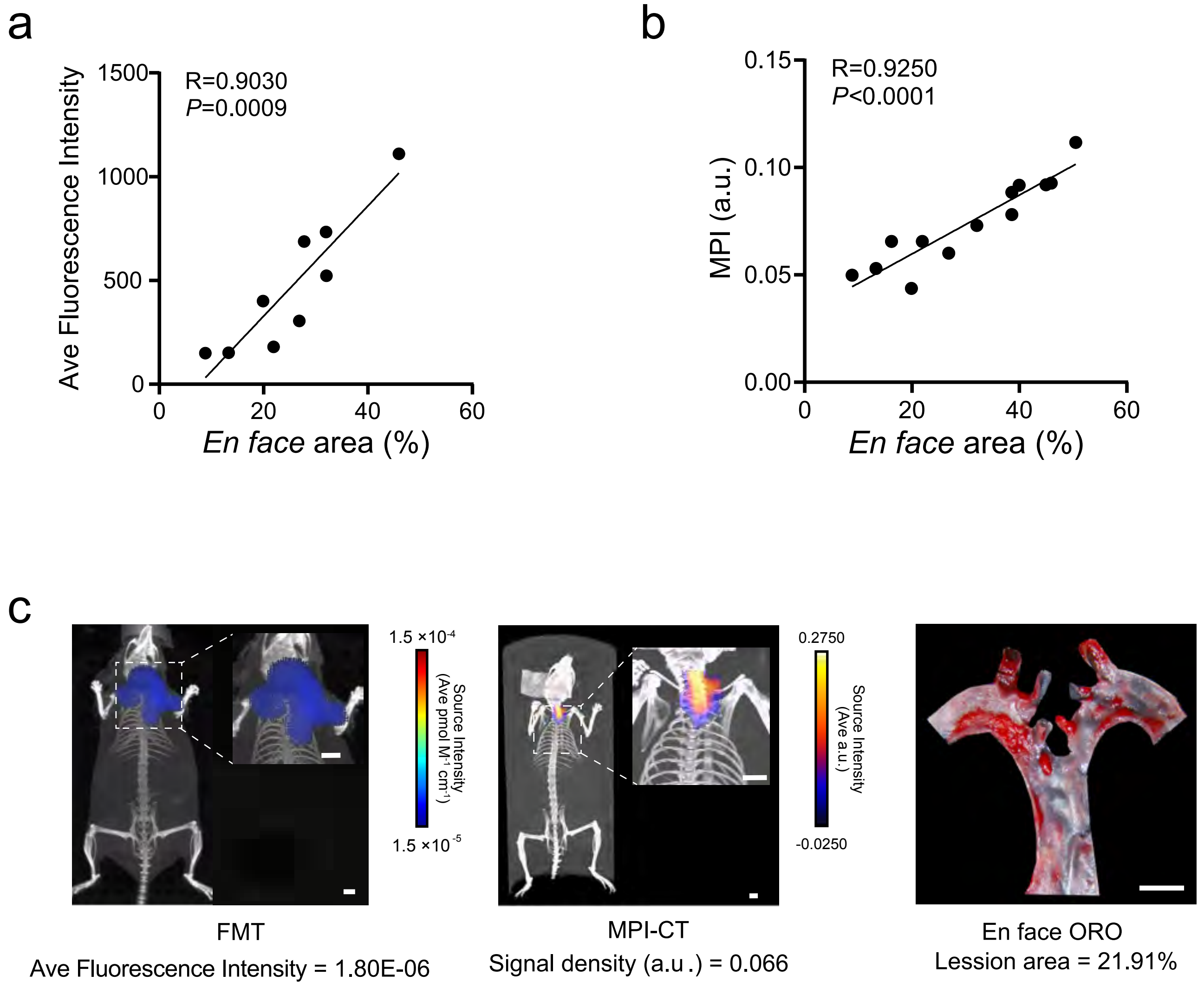
a
R=0.9030
P=0.0009
Ave Fluorescence Intensity
1500
1000
500
0
0
20
40
60
En face area (%)
b
R=0.9250
P<0.0001
MPI (a.u.)
0.15
0.10
0.05
0.00
0
20
40
60
En face area (%)
c
1.5 ×10-4
Source Intensity
(Ave pmol M-1 cm-1)
1.5 ×10-5
0.2750
Source Intensity
(Ave a.u.)
-0.0250
FMT
Ave Fluorescence Intensity = 1.80E-06
MPI-CT
Signal density (a.u .) = 0.066
En face ORO
Lession area = 21.91%

Figure S6

Figure S6. *In vivo* and *ex vivo* tracking of atherosclerotic plaque formation

(a) Correlation between atherosclerosis assessed by *En face* ORO staining and *in vivo* FMT imaging using 5HFeC NPs probe.
(b) Correlation between atherosclerosis assessed by *En face* ORO staining and *in vivo* 3D-MPI imaging using 5HFeC NPs probe.
(c) Validation of atherosclerotic plaque formation after pathogenic induction using 3 independent approaches. Scale bar=2mm.
